# Supplementary material for: How did we protect children against COVID-19 in Iran? Prevalence of COVID-19 and vaccination in the socio-economic context of COVID-19 epidemic
Source: PLoS One. 2024 May 9;19(5):e0303043. doi: 10.1371/journal.pone.0303043 (PMC11081304; doi:10.1371/journal.pone.0303043)
Supplement: Attachment — Submitted filename: response to reviewers.docx [file pone.0303043.s002.docx]

**Dear Nour Amin Elsahoryi;**

Thank you for giving us the opportunity to submit a revised draft of our manuscript titled " How did we protect children against COVID-19 in Iran? Prevalence of COVID-19 and vaccination in the socio-economic context of an epidemic" to PLOS ONE.

We appreciate the time and effort you and the reviewers have dedicated to providing valuable feedback on our manuscript. We are grateful to the reviewers for their insightful comments on our paper. We have been able to incorporate changes to reflect most of the suggestions provided by the reviewers. We have corrected them on the manuscript and responded to all comments here.

**A- Response to the Editor**

**Comment**

Please ensure that your manuscript meets PLOS ONE's style requirements, including those for file naming.

**Response**: The final version of the manuscript was checked and submitted according to the instructions.

**Comment**

"We suggest you thoroughly copyedit your manuscript for language usage, spelling, and grammar. If you do not know anyone who can help you do this, you may wish to consider employing a professional, scientific editing service"

The name of the colleague or the details of the professional service that edited your manuscript

**Response:** Javad Ghholami, Virayeshyar English language editing and Translation center

**Comment**

Please provide an amended statement that declares all the funding or sources of support

**Response:** This study sponsored by the University of Social Welfare and Rehabilitation Science [contract number: 1401/801/A/11799 ] (https://uswr.ac.ir/) received by Meroe Vameghi and Alborz University of Medical Sciences, Karaj, Iran [contract number: 1401/60/D/1495] (https://abzums.ac.ir/) received by Giti Bahrami. Moreover, no additional external funding was received for this study.

**Comment**

PLOS requires an ORCID iD for the corresponding author in Editorial Manager on papers submitted after December 6th, 2016.

**Response: ORCID id of Dr. Mohammad Saatchi: 0000-0003-2744-9927**

**Unfortunately, we cannot log in to ORCID account on the first page of the Editorial Manager. We face this error:**

**B- Response to the Reviewers' comments:**

**Reviewer #1:**

**However, it needs a thorough copy editing. I found the following errors, some of which are clear errors, while others are simply ambiguities. Errors must be corrected before the paper is published. Raw data is not provided, so it is not possible to check the data, but I strongly urge that the authors recheck key statistics. A correction or retraction would be detrimental to the authors themselves, as well as calling into question the conclusions in the paper.**

Author: We checked all data again.

**66>> Some references related to evidence of support for the paper's assumptions were to opinion pieces, not to research articles. (9 and 10 for example). References to support statements of fact should be to research that contains that evidence.**

Author: Using Opinions of government officials instead of research findings is due to a lack of governmental official reports and any research results on many aspects of the COVID-19 pandemic in Iran, including the prevalence of COVID-19 infection, vaccination, and vaccine hesitancy among children.

**74>>"Feb 2019" as start of epidemic is wrong**

Author: Feb 2019 was corrected to Feb 2020

**232>>The report of definitely unwillingness among Iranians living in Tehran and Karaj was 65% and 29%, respectively, and among Afghans, it was 41% and 24%
>> "definite unwillingness" should be clarified. The figure is missing important axis and legend information.**

Author: There is no definition for " definite unwillingness ", but as an option of a person's desire to receive a vaccine in the future, it indicates that people, according to their feelings, understanding, and existing conditions, not only have not received a vaccine so far, but in the future, they definitely do not want to receive the Covid-19 vaccine.

**307>>it's—its**

Author: It was corrected to its

**415>> In Iran, some studies and reports have predicted an escalation of financial conditions following the onset of the pandemic. “escalation" does not make sense in this context. Escalation would be an increase. It should be a "worsening" or an "escalation in poverty".**

Author: “Escalation” was corrected to “worsening”

**437>> The government's failure to adequately meet the financial needs of the people during the pandemic made it less strict about implementing preventive measures, such as the closure of jobs and using masks, which led to an increase in COVID-19 infection among children and adults. >> No support for how a failure to meet financial needs made it less strict about implementing preventive measures. Meaning unclear.**

Author: This means that because the government could not support businesses' closure during the pandemic, it allowed businesses to remain open despite the high prevalence of COVID-19. This situation led to an increase in COVID-19 infection among children and adults. We corrected this part with clearer phrases.

**Reviewer#2**

**1>> This should be a research question and should be placed in the methodology section.**

Author: We add this to method section.

**2>> COVID-19 epidemic in Iran**

Author: We changed the title based on the comment

**27>> Mention its version number**

Author: We add the version number

**104> Did the study cover all districts in both sites?**

Author: Yes. As we explained, 22 districts of Tehran and 12 districts of Karaj were investigated using cluster sampling.

**106>> What was your unit of sampling? It is a household.**

Author: Exactly. In each cluster, the household was the study unit. It added.

**134>> House ownership**

Author: It changed to house ownership

**176> Your observation was 2878. But it is not for instance in the case of child sex, etc**

**Why?**

Author: The missing data for each of the variables in the study makes the number of variables not equal to the total number.

**181>>Income = 98.3% Child’s needs = 98% Why not 100%?**

Author: We couldn’t understand the comment

**190>> Table 2**

Author: All comments for Table 2 regarding the difference between the numbers of the total sample of study and the numbers in the table are because of missing data

**213>> 61% + 41% = 102%, why?**

Author: These two values should not be added together. One is the percentage for Tehran, and the other is the percentage for Karaj. In total, 54% of participants have received at least one dose of vaccine.

**214>> 42% + 83.2% = 125.2%?**

Author: These two values should not be added together. One is the percentage of vaccination coverage in the 7-14 age group, and the other is the percentage of vaccination coverage in 15-18 age group

**231>>1206 in table 3.**

Author: It is corrected

**279>> To say representative, the target and study populations would have been indicated. You only indicated the general sampled study population. The target population was not indicated. Since it was randomly sampled, I think it is representative.**

Author: The target population in the present study was families with children under 18 years of age in Tehran. Using random sampling, we tried to select a representative sample of these households.

**335>> What is the immunological reason for this?**

Author: We don’t consider the causes of this difference according to immunological reasons, but rather deciding to no vaccinating children is the result of harm estimation for infection by covid-19 and vaccination out comes for two age groups.

**345>> Is it to mean similar findings?**

Authors: Yes, it is, and it is corrected.

**359>> How?**

Author: According to some societal norms, vaccination could not change the predetermined death for people. We changed the sentence to: “Social norms (such as the concept of “fatalism” expresses the belief that everything that happens in life is predetermined, it is not possible to go beyond this predetermination) (Erkal et al, 2023)”

388>> Why? What is the Immunological mechanism involved?

Author: We added the immunologic reasons to the paper, but it seems somehow unrelated to a discussion based on a more sociologic interpretation of findings, and we prefer not to add this part to the paper.

“According to a recent review study, key mechanisms that protect young children from severe COVID-19 disease include the placental barrier, differential expression of angiotensin-converting enzyme 2 (ACE-2) receptors, immature immune response, and passive transfer of antibodies via placenta and human milk.”
